# Role of Radiofrequency Ablation in the Management of Unresectable Pancreatic Cancer

**DOI:** 10.3389/fmed.2020.624997

**Published:** 2021-02-11

**Authors:** Muhammad Nadeem Yousaf, Hamid Ehsan, Ahmad Muneeb, Ahsan Wahab, Muhammad K. Sana, Karun Neupane, Fizah S. Chaudhary

**Affiliations:** ^1^Department of Medicine, MedStar Union Memorial Hospital, Baltimore, MD, United States; ^2^MedStar Franklin Square Medical Center, Baltimore, MD, United States; ^3^MedStar Good Samaritan Hospital, Baltimore, MD, United States; ^4^MedStar Harbor Hospital, Baltimore, MD, United States; ^5^Department of Medicine, Faisalabad Medical University, Faisalabad, Pakistan; ^6^Department of Medicine, Baptist Medical Center South, Montgomery, AL, United States; ^7^Department of Medicine, King Edward Medical University, Lahore, Pakistan; ^8^Department of Medicine, Manipal College of Medical Sciences, Pokhara, Nepal

**Keywords:** pancreatic cancer, radiofrequency ablation, endoscopic ultrasound, endoscopic retrograde cholangiopancreatography, palliative cancer care

## Abstract

Pancreatic cancer is one of the most aggressive malignancies of the digestive tract and carries a poor prognosis. The majority of patients have advanced disease at the time of diagnosis. Surgical resection offers the only curative treatment, but only a small proportion of patients can undergo surgical resection. Radiofrequency ablation (RFA) is a well-known modality in the management of solid organ tumors, however, its utility in the management of pancreatic cancer is under investigation. Since the past decade, there is increasing use of RFA as it provides a feasible palliation treatment in the management of unresectable pancreatic cancer. RFA causes tumor cytoreduction through multiple mechanisms such as coagulative necrosis, protein denaturation, and activation of anticancer immunity. The safety profile of RFA is controversial because of the high risk for complications, however, small prospective and retrospective studies have shown promising results in its applicability for palliative management of unresectable pancreatic malignancies. In this review, we discuss different approaches of RFA, their indications, technical accessibility, safety, and major complications in the management of unresectable pancreatic cancer.

## Introduction

Pancreatic cancer is one of the most aggressive gastrointestinal malignancies and is the fourth leading cause of mortality in the United States despite advancement in both diagnostic and therapeutic interventions in the management of these patients. Annual incidence of pancreatic cancer in the United States is ~57,600 cases, while estimated mortality rate is 47,050 with a slight male predominance (1). Pancreatic cancer has a poor prognosis with a 5 years relative survival rate of only 9% (1). Surgical resection provides the only potential curative option in pancreatic cancer patients (2). However, only 15–20% of the patients with pancreatic cancer are eligible for surgical resection, as majority of them present with locally advanced stages or with distant metastasis when surgical resection is not possible (3). A multimodality approach is required in the management of pancreatic cancer even in patients undergoing surgical resection for curative intent. A multimodal approach involves the systemic chemotherapy (adjuvant/neoadjuvant), local ablation and surgical resection (depending upon the staging of pancreatic tumor) (4). Adjuvant treatment is an important part of management in those patients who have undergone surgical resection since the 5-year survival rate in these patients is only around 20% (4). Patients with unresectable pancreatic cancer have a median survival of only 11–15 months after chemoradiation (3, 5). In comparison, the survival rate extends to 22–26 months after surgical resection, adjuvant or neoadjuvant chemotherapy (3, 5). For patients with unresectable pancreatic cancer, palliation with chemoradiation therapy and endoscopic interventions are utilized to improve quality of life. However, these palliative treatments barely change the outcome of disease. In addition to chemoradiation, various modalities (matrix metalloproteinases, targeted therapies, angiogenesis inhibitors, epidermal growth factor receptor inhibitors, and immunotherapies) are emerging for the treatment of patients with pancreatic adenocarcinoma (4). Radiofrequency ablation (RFA) is a well-known modality that has been used effectively for the treatment of solid tumors, such as hepatocellular cancer, cholangiocarcinoma, malignancies involving lungs, breast, kidney, bones, and prostrate (6, 7). RFA has been found to be superior to percutaneous ethanol injection with overall survival rates of 55% vs. 42%, respectively (*p* < 0.01) in patients with unresectable stage I–II hepatocellular carcinoma (8). RFA has also been found to be effective in the treatment of intrahepatic cholangiocarcinoma of 3 cm or less with complete necrosis seen in 100% patients (9). However, the definitive role of RFA for pancreatic cancer remains under investigation. Implementation of RFA in the management of unresectable pancreatic cancer is a relatively newer treatment option that may potentially provide an effective palliation in these patients due to cytoreduction of tumor (10). In addition to thermal effect, it is proposed that RFA triggers antitumor immunity by activating cancer specific T lymphocytes and heat shock protein-70 (11, 12). The efficacy and safety of RFA procedure is unclear in literature. In this review, we provided an overview of RFA and discussed various approaches of RFA therapies in the management of pancreatic cancer. To identify the relevant published literature, we performed a comprehensive search on PubMed, Google Scholar, Cochrane, Clinicaltrials.gov, and browsed through the references of relevant studies using the MeSH terms “pancreatic cancer” and “radiofrequency catheter ablation.”

## Principles and Protocol of Radiofrequency Ablation Therapy

Radiofrequency ablation involves delivery of thermal energy to the tumor through special needle electrodes that leads to coagulative necrosis with protein denaturation and decreased tumor bulk (13). Human cells cannot withstand temperature above 50°C and start undergoing denaturation. Temperature as high as 60°C results in cell death (14). Application of the high frequency alternating current (200–1,200 kHz frequency) via an electrode causes an agitation of positive and negatively charged ions within the tissue and produce additional heat due to friction. Heat production is maximum in the area around the electrode because of a high flow of electrical current. This heat energy results in coagulative necrosis of the tumor eventually leading to reduction of tumor volume (15, 16). The protocol to use RFA therapy in the management of hepatocellular or cholangiocarcinoma is well-established in the current practice guidelines (17). Such protocol to use RFA therapy for pancreatic cancer does not exist in the current practice guidelines because of lack of sufficient data. Current use of RFA in pancreatic cancer is based on the individual experiences of expertise and medical center specific protocols. Precise control of temperature, frequency of current, and duration of the delivery of alternating current is crucial as uncontrolled heat can lead to excessive charring resulting in circuit break. In the event of large tumor bulk, charring can be controlled with the use of saline irrigation (18). A recommended safe temperature for RFA is 90°C (mean) as temperature higher than 105°C results in increased risk of adverse events without favorable impact on tumor size.

## Radiofrequency Ablation Techniques

RFA of pancreatic tumors can be performed using different approaches, that include the intraoperative approach, percutaneous approach under ultrasound or radiologic imaging guidance, an endoscopic approach using endoscopic ultrasound (EUS) or endoscopic retrograde cholangiopancreatography (ERCP). RFA poses a risk of potential adverse events both to surrounding vital structures as well as pancreas itself. Common potential adverse events associated with RFA therapy are acute pancreatitis, pancreatic fistula, gastrointestinal hemorrhage, sepsis, portal vein thrombosis, and damage to surrounding structures, such as duodenum or bile duct (19). However, the risk of these adverse events is low with modifications of RFA techniques, such as altering ablation parameters like ablation temperature, distance of RFA needle from adjoining structures, and introducing other safety measures like duodenal and inferior vena cava cooling during ablation (20–22).

## Intraoperative Radiofrequency Ablation

### Indications and Technical Accessibility

Intraoperative RFA is indicated in unresectable, non-metastatic and locally advanced pancreatic tumor involving pancreatic head or uncinate process that results in either obstructive jaundice or gastric outlet obstruction. It is also performed in patients who are found to be inoperable during surgery or those who are not amenable to percutaneous imaging guided or endoscopic guided interventions for palliation (7, 23–25). Intraoperative RFA involves thermal ablation of tumor during laparotomy. If tumor involves pancreatic head, Kocher maneuver is performed to expose head of the pancreas. Continuous cooling is used to prevent thermal damage to the surrounding structures. For cooling of duodenum, a nasogastric tube is placed in the proximal duodenum and cold saline is irrigated continuously. Cold gauze can be placed over inferior vena cava to protect it from thermal injury. RFA needle is inserted under ultrasonographic guidance during surgery to avoid damage to the nearby vital structures. Thermal energy is delivered after positioning the specialized RFA needle in the middle of the tumor. A safe needle distance from the duodenum and other surrounding structures should be maintained to prevent thermal damage to these structures (7, 25, 26).

### Safety and Adverse Events

Hlavsa et al. compared 24 patients with intraoperative RFA (intervention group) with 24 patients who underwent only surgical bypass procedure and reported lower rate of 3 months mortality of 16.6 vs. 41.7%, comparable morbidity of 8.3%, and relatively higher overall median survival 9.9 vs. 8.3 months in RFA group compared with control group (*p* = 0.758) (25). Median survival was better among patients with grade I and II tumors after RFA than grade III tumor (25). Although results of this study did not show significant survival benefits, however, RFA appears to be feasible palliative option in well-differentiated unresectable pancreatic cancer. In a small study of 4 patients with locally advanced unresectable pancreatic cancer, no difference of survival was noted after intraoperative RFA, however, CA 19-9 tumor marker was decreased in all patients without adverse events at 12 months follow up (7). Zou et al. used a modified technique with a combination of an intraoperative RFA and implantation of radioactive iodine (I^125^) seed within the pancreatic tumor in 32 patients that resulted in the improvement in quality of life with a decreased median pain score (from 5.86 ± 1.92 to 2.65 ± 1.04) at 1 month and an increased survival time upto 17.5 months that was longer for stage III cancer as compared to stage IV cancer (27). A combined complete or partial regression of tumor was noted in 78.1% of patients, while 15.6% patients did not respond to this approach (27).

Common adverse events associated with intraoperative RFA are gastrointestinal bleeding, acute pancreatitis, biliary or pancreatic duct fistula, biliary leak, and post-operative wound or intra-abdominal infections. Matsui et al. used intraoperative RFA in 20 patients with high technical and clinical success of procedure as decrease in serum tumor markers was found in 14 patients and two patients experienced serious adverse events, such as septic shock and gastrointestinal hemorrhage (23). Varshney et al. reported partial necrosis (up to 3 cm) of the tumors with RFA in three patients with inoperable pancreatic adenocarcinoma and minor self-limiting adverse events in two patients (10). Wu et al. assessed the safety of cool tip RFA in 16 pancreatic cancer patients and recommended a distance between RFA site and major peripancreatic vessels should be >5 mm as mortality rate of 25% was noted in patients with tumor closer to portal vein (24). In a study of 50 patients, 30 days mortality rate was only 2% with intraoperative RFA and a significant reduction of procedure related complications was noted by decreasing RFA temperature from 105 to 90°C. In this study only 6/50 patients experienced RFA related adverse events, such as pancreatic fistulas (two patients), portal vein thrombosis (four patients), duodenal bleeding (two patients), and pancreatitis (one patient) (22). In a larger study of 265 patients, overall morbidity and mortality were 23.4% (62/265) and 1.5% however, a higher rate of RFA-related adverse events 12.8% (34/265) was found as compared to overall surgical adverse events 10.4% (28/265). Overall survival, disease-specific survival and progression-free survival of first 200 patients as reported by an interim analysis were, 19, 19, and 13 months, respectively (28, 29).

## Percutaneous Radiofrequency Ablation

### Indications and Technical Accessibility

Percutaneous RFA is indicated in selected number of patients with locally advanced and unresectable pancreatic cancer without evidence of metastasis. Percutaneous RFA is a minimally invasive technique that involves percutaneous passage of RFA needle into malignant lesion under guidance of an abdominal ultrasound or radiological imaging, such as CT scan which is performed before the procedure to assess the accessibility of the lesion and technical feasibility of the procedure. After confirming the potential route of RFA needle, it is advanced into the lesion. Effort is made to avoid damage to the adjacent blood vessels and surrounding structures. RFA electrodes are then positioned in the center of the tumor and thermal energy is delivered for ablation of tumor. A real time monitoring of thermal effect of RFA on tumor and surrounding structures can be seen with ultrasound. Ablation time, power and other parameters are adjusted according to the tumor size and tissue impedance (30).

### Safety and Adverse Events

In a small pilot study of eight patients with neuroendocrine unresectable pancreatic cancer, ultrasound guided percutaneous RFA was performed in seven patients and a high clinical success of procedure as tumor regression was noted in all patients on median follow up of 34 months without any mortality (31). Similar results of safety and feasibility of CT scan-guided RFA was reported in several studies (Table 1) (30–35). D'Onofrio et al. assessed the feasibility and effectiveness of percutaneous RFA in 18 patients with non-metastatic unresectable pancreatic adenocarcinoma and achieved a technical success of 93% in 16 out of 18 patients with a mean survival of 185 days (range 62–398 days) (30). The tumor size remained stable in 55.6% (10/18) of patients at 1 month of follow up abdominal CT scan, and increased in 44.4% (8/18) patients which raised question about the effectiveness of percutaneous RFA (30). Mizandari et al. performed percutaneous intraluminal RFA coupled with stent placement was used in 134 patients with malignant obstructions of bile and pancreatic ducts (32 patients with pancreatic adenocarcinoma) and reported a 97% success rate of procedure with only two patients experienced procedural technique related adverse events (contrast extravasation) following RFA (36).

**Table 1:** Studies demonstrating the feasibility of percutaneous ablation therapies for pancreatic cancer.

**References**	**Total patients (*n*)**	Age^*^	**No. of tumors (*n*)**	**Type of tumor**	Size of tumor^*^ **(mm)**	**Approach of PC ablation**	**Probe size**	**Clinical outcome *n* (%)**	**Adverse events (*n*)**	Follow up^*^ **(months)**
Carrafiello et al. (32)	1	77	1	Pancreatic metastasis	20	PC with CT	19 G	1/1 (100%), Complete resolution of lesions	Abdominal pain, Hyperamylasemia	12
Limmer et al. (33)	1	80	1	Insulinoma	15	PC with CT	16 G	1/1 (100), Complete resolution of symptoms and lesions	None	1.25
Wu et al. (34)	1	64	1	Gastrinoma	40	PC with CT	NA	1/1 (100%) improvement of symptoms with reduction in primary and metastatic lesions	none	20
Singh et al. (35)	1			PDAC		PC with CT	NA	1/1 (100%) Partial reduction in tumor size (up to 3cm)	None	NA
Rossi et al. (31)	7	62	1	PNET	16	PC with CT	17 G 19 G	6/7 reduced tumor size, 1/7 complete resolution	Increased serum amylase and lipase within 24 h (6); Pancreatitis (1)	34
D'Onofrio et al. (30)	18	62.4	18	PDAC	48.1	PC with US	17 G	8/18 (44.4%) increase in tumor size 10/18 (55.6%) stable lesions at 1 month	None	6

*PDAC, pancreatic ductal adenocarcinoma; PNET, pancreatic neuroendocrine tumor; PC, percutaneous; US, ultrasound; CT, computed tomography; NA, not available*.

* *Mean*.

## Endoscopic-Guided Radiofrequency Ablation

### Indications and Technical Accessibility

EUS and fluoroscopic-guided radiofrequency ablation (RFA) can also be used to ablate locally advanced neoplastic lesions that have not yet metastasized (37, 38). In the past decade, there is increasing use of endoscopic-guided RFA for unresectable pancreatic adenocarcinoma, resectable tumors in patients that cannot undergo surgery or chemotherapy because of comorbidities and those patients who are not responsive to other therapies (39, 40). Endoscopic-guided RFA is also a minimally invasive approach that involves positioning of duodenoscope in the stomach or duodenum closer to the pancreatic tumor and passage of an electrode needle into the tumor under endoscopic guidance for tumor ablation. This technique involves the application of a high-frequency probe around the malignant tissue, causing coagulative necrosis from radiofrequency-induced hyperthermia. Specifically, for pancreatic cancer, commercially available RFA probes are available that are advanced over 0.035-inch guidewire through a specialized catheter compatible with standard ERCP or EUS duodenoscope (41). Endoscopic-RFA is commonly used for the treatment of stage III pancreatic adenocarcinoma and should be considered in the management of locally advanced or unresectable pancreatic cancers in the absence of distant metastases (42). It has been used as initial management at the time of diagnosis, as combined therapy and in case of failure of standard systemic treatment options (13, 38, 43). Stage IV patients have also been included in a few studies with some benefit (24, 44).

Care is taken during insertion of RFA probe to avoid damage to normal parenchyma and surrounding structures including pancreatic or bile duct and major blood vessels adjacent to tumor. The needle tip is placed at the distal end inside the tumor. After confirmation of the needle position with EUS, thermal energy is delivered. In case of larger lesions, position of electrode may be changed under EUS guidance in order to ablate other areas within the lesion. Application of RFA may cause visual obscurities, therefore, it is advisable to ablate the technically challenging part of the tumor first (45). The recommended thermal energy for effective tumor ablation ranges from 60 to 100°C as temperature >100 may result in a higher risk of adverse events due to damage to surrounding structures (19). In addition to fragile pancreatic parenchyma that can be damaged by high temperatures, several anatomic challenges may hinder the use of RFA in the treatment of pancreatic cancer. These include the retroperitoneal location of the pancreas, a close relation of the pancreas to the duodenum, stomach, transverse colon and portal vein and involvement of the bile duct. Thus, there is a substantial risk of thermal damage to these structures if RFA is used for the pancreatic cancer (46). To avoid thermal damage to the surrounding vital structures, a circular area is spared at the tumor margins (47). Complete ablation of tumors located near large blood vessels is challenging because of the cooling effect generated by the blood flow (38). During the procedure, RFA-electrodes are positioned around the neoplastic tissue under direct visualization with an endoscope, thus minimizing the risk of damage to the adjacent tissues and blood vessels (38). Direct ablation of the entire tumor may not be feasible in cases of retroperitoneal extension and vascular invasion of the pancreatic tumor (46). Ablation may also prove to be difficult during laparotomy, particularly if liver metastases are found that were not detected before procedure (46).

RFA with subsequent stent placement has been successfully used to re-canalize biliary or pancreatic ducts that were obstructed by unresectable tumors (36). Indeed, ductal decompression with stenting is considered standard of care in patients with malignant obstruction of biliary or pancreatic ducts due to unresectable tumors, however, stents are often prone to occlusion (23, 24, 48–50). When RFA is combined with stenting, specifically in these circumstances, stent patency is prolonged, presumably by reducing tumor volume and due to immunomodulatory effects, halting tumor regrowth (50–52). Though RFA combined with stenting is safe and prolongs stent patency, reports on the mortality benefits of this combination are conflicting (53, 54). Preoperative abdominal CT-scans are considered to be the standard of care in order to determine the exact location of the tumor, its dimensions, the presence or absence of abdominal metastasis and vascular invasion (55). Though there are multiple approaches to access the pancreas including transgastric or transduodenal endoscopy, open laparotomy or percutaneous approach, an endoscopic approach remains the most feasible and minimally invasive approach and has been shown to provide superior outcomes (56).

### Safety and Adverse Events

Endoscopic-RFA for unresectable pancreatic cancer is a relatively safer approach with a high technical and clinical success rate and less risks of procedure-related mortality and adverse events (Table 2) (39, 40, 57–71). A recent meta-analysis of 14 studies with 158 patients has shown a pooled clinical success rate of EUS-RFA 83.5% [95% confidence interval (CI) 67.9–92.4%] while adverse events rate of 32.2% (95% CI 19.4–48.4%) with majority of adverse events managed medically (72). In another large meta-analysis of 13 studies with 127 patients, Dhaliwal et al. demonstrated a very high pooled technical success rate (98%), pooled clinical success rate (84.5%) and safety profile of EUS-RFA in the management of unresectable pancreatic cancer (73). In this meta-analysis, the overall adverse events rate 1 week after EUS-RFA was 13.4%, with commonly reported adverse events being abdominal pain 8.81% (95% CI, 2.72–16.88) followed by bleeding and pancreatitis observed in 1 patient each while perforation or procedure-related infections were not reported in any of the patients (73). Multiple small prospective and retrospective studies have shown promising results of EUS-RFA safety, its clinical and technical success as compared to intraoperative and percutaneous RFA (38–40, 60, 67, 74–76).

**Table 2:** Studies demonstrating the efficacy and safety of endoscopic ultrasound guided ablation therapies for unresectable pancreatic cancer.

**References**	**Total patients (*n*)**	**Age (mean)**	**No. of tumors (*n*)**	**Types of pancreatic tumor**	**Size of tumor (mm) (mean)**	**Approach of EUS Ablation**	**Ablation sessions/ lesions**	**Technical success *n* (%)**	**Clinical success** ***n*** **(%)**		**Adverse events (*n*)**	**Follow up (mean months)**
									**Resolution of symptoms**	**Resolution/ decrease in tumor size**		
Arcidiacono et al. (57)	16	^*^61.9	16	LAPDAC	35.7	EUS-RFA	16/16	16/22 (73)	–	6/6 (100)	Abdominal pain (3), Bleeding (1), Hyperamylasemia (1), Obstructive jaundice (2), Duodenal strictures (1)	6
Levy et al. (58)	5	66	5	Insulinoma	15	EUS-EA	11/5	5/5 (100)	5/5 (100)	NA	None	13
Pai et al. (59)	7	^*^69	7	PDAC	35.2	EUS-RFA	3^*^	7/7 (100)	–	7/7 (100)	Pancreatitis	3–6
Wang et al. (60)	3	62.7	3	Unresectable pancreatic cancer	37.3	EUS-RFA	4/3	3/3 (100)	3/3 (100)	3/3 (100)	None	1.5
Park et al. (61)	11	52.5	14	NNET (9), Insulinoma (2)	12.3	EUS-EA	18/14	11/11 (100)	2/2 (100)	13/13 (100)	Abdominal pain (1), Pancreatitis (3), Pancreatic duct stenosis (1).	12
Song et al. (39)	6	^*^62	6	LAPDAC	38	EUS-RFA	8/6	6/6 (100)	–	6/6 (100)	Abdominal pain (2)	2–6
Lakhtakia et al. (62)	3	45	6	Insulinoma	–	EUS-RFA	9/3	3/3 (100)	3/3 (100)	2/3 (67)	None	11–12
Paik et al. (63)	8	^*^55	8	NNET (2), Insulinoma (3), Gastrinoma (1), SPN (2)	15	EUS-EA	8/8	8/8 (100)	4/4 (100)	6/8 (75)	Abdominal pain (2)	16.5
Qin et al. (64)	7	NA	7	Insulinoma	8–34	EUS-LI	11/11	11/11 (100)	7/7 (100)	NA	None	1–18
Di Matteo et al. (65)	9	^*^74.7	09	LAPDAC	35.4	EUS-LA	9/9	9/9 (100)	–	9/9 (100)	Pseudocyst (3), Hyperamylasemia (2)	7.4
Crino el al. (40)	8	67	08	LAPDAC (7), Metastatic RCC (1)	36	EUS-RFA	12/8	8/8 (100)	–	8/8 (100)	Abdominal pain (3), Hyperamylasemia (1)	6
Choi et al. (66)	10	21–71	10	NNET (7), Insulinoma (1), SPN (2)	20	EUS-EA	16/10	10/10 (100)	1/1 (100)	10/10 (100)	Abdominal pain (1), Pancreatitis (1)	42
Scopelliti et al. (67)	10	^**^50–71	10	LAPDAC	25–75	EUS-RFA	14/10	10/10 (100)	–	9/10 (90)	Asymptomatic ascites (2), Peripancreatic effusion (2)	1
Barthet el al. (68)	12	59.9	14	NNET	13.1	EUS-RFA	–	12/12 (100)	–	12/14 (87.5)	Bacteremia (1), Pancreatic duct stenosis (1)	12
Oleinikov el al. (69)	18	60.4	27	NNET (11), Insulinoma (7)	14.3	EUS-RFA	–	26/27 (96.2)	7/7 (100)	25/27 (92.5)	Pancreatitis (2)	2–21
Matsumoto et al. (70)	5	^**^55–74	5	NNET	10	EUS-EA	8/5	5/5(100)	–	4/5 (80)	None	12
Oh et al. (71)	13	^*^60	13	Pancreatic serous cystic neoplasms	50^*^	EUS-RFA	19/13	13/13 (100)	13/13 (100)	8/13 (61.5)	Abdominal pain (1)	9.21^*^

*NA, not available; LAPDAC, locally advanced pancreatic ductal adenocarcinoma; NNET, non-functional neuroendocrine tumor; SPN, serous pancreatic neoplasm; H, head; BNT, body neck and tail; EUS, endoscopic ultrasound; RFA, radiofrequency ablation; EA, ethanol ablation; LI, lauromacrogol injection; LA, laser ablation*.

* Median.

** *Range*.

Radiofrequency hyperthermia has shown improvement in the palliation and response to the treatment by reducing the requirement of a high dose of chemotherapy (74, 77). Immense heating of the surrounding structures of the tumor, rather than damage caused by the tip of RFA probe, is associated with adverse events (55). Common adverse events of RFA are gastrointestinal hemorrhage, biliary leakage, duodenal injury, portal vein thrombosis and sepsis, while damage to normal pancreatic tissue may result in pancreatic ascites, pancreatic fistula, necrotizing pancreatitis and pseudocyst formation (22, 42, 55). High morbidity (0–40%) and mortality (0–25%) rates were reported in the early phase of RFA application for pancreatic cancer (78). Later studies have shown fewer adverse events if the temperature and length of the dispensed energy are adjusted (79). It has been suggested that RFA temperature of 90°C causes fewer adverse events as compared to higher temperatures (22, 79, 80). Probe distance of 10 mm from the duodenum and 15 mm from the portal and mesenteric vessels is recommended (20, 79). Continuous cooling of the duodenum using 100 ml/min saline at 5°C is also beneficial in reducing duodenal adverse events (20, 81). Some adverse events can also be reduced if gastric and biliary bypass procedures are performed concurrently (46). Taken together, EUS-RFA is a relatively safer modality and adjunct to chemotherapy and standard multidisciplinary management of unresectable pancreatic cancer. Multiple small studies have shown its safety because of high clinical success and less risk of procedure-related mortality and adverse events. However, there is a lack of data on improvement in the quality of life with the utility of RFA that prompts need for large randomized controlled trials to assess the efficacy of this modality in the management of unresectable pancreatic cancer.

## Conclusions

Radiofrequency ablation has been increasingly applied in the management of unresectable pancreatic cancer. Both intraoperative and percutaneous RFA have shown the acceptable clinical and technical success rate, however clinical safety and risks of serious adverse events is concerning. With the development of more effective chemotherapy regimen and recent advancement of endoscopic devises, application of endoscopic RFA has shown promising results in the palliation of unresectable pancreatic cancer. EUS-RFA is relatively safer than intraoperative and percutaneous approach with a higher clinical and technical success rate and less risk of adverse events. Currently, large prospective studies to assess long term impact of RFA on quality of life and survival are lacking. This warrants the need for prospective clinical trials in the future to validate its role in pancreatic cancer.

## Author Contributions

MNY: manuscript writing, overall data collection, supervision, review, and revision. HE, AM, and FC: manuscript writing and overall data collection. AW: manuscript writing, review, and revision. MS and KN: literature search and data collection. All authors contributed to the article and approved the submitted version.

## Conflict of Interest

The authors declare that the research was conducted in the absence of any commercial or financial relationships that could be construed as a potential conflict of interest.
